# Transmembrane helix interactions regulate oligomerization of the receptor tyrosine kinase EphA2

**DOI:** 10.1016/j.jbc.2024.107441

**Published:** 2024-06-03

**Authors:** Daniel Wirth, Ece Özdemir, William C. Wimley, Elena B. Pasquale, Kalina Hristova

**Affiliations:** 1 Department of Materials Science and Engineering, Johns Hopkins University, Baltimore, Maryland, USA; 2 Department of Biochemistry and Molecular Biology, Tulane University School of Medicine, New Orleans, Louisiana, USA; 3 Cancer Metabolism and Microenvironment Program, Sanford Burnham Prebys Medical Discovery Institute, La Jolla, California, USA

**Keywords:** single-pass membrane receptor, receptor tyrosine kinase, EphA2, transmembrane domain, oligomerization

## Abstract

The transmembrane helices of receptor tyrosine kinases (RTKs) have been proposed to switch between two different dimeric conformations, one associated with the inactive RTK and the other with the active RTK. Furthermore, recent work has demonstrated that some full-length RTKs are associated with oligomers that are larger than dimers, raising questions about the roles of the TM helices in the assembly and function of these oligomers. Here we probe the roles of the TM helices in the stability of EphA2 RTK oligomers in the plasma membrane. We employ mutagenesis to evaluate the relevance of a published NMR dimeric structure of the isolated EphA2 TM helix in the context of the full-length EphA2 in the plasma membrane. We use two fluorescence methods, Förster Resonance Energy Transfer and Fluorescence Intensity Fluctuations spectrometry, which yield complementary information about the EphA2 oligomerization process. These studies reveal that the TM helix mutations affect the stability, structure, and size of EphA2 oligomers. However, the effects are multifaceted and point to a more complex role of the TM helix than the one expected from the “TM dimer switch” model.

Receptor tyrosine kinases (RTKs) are single-pass transmembrane proteins that control cell growth, differentiation, motility, and metabolism (1, 2, 3). They transduce biochemical signals *via* lateral oligomerization in the plasma membrane. The catalytic activity of the intracellular kinase domains is stimulated by cross-phosphorylation of neighboring RTK molecules assembled in dimers and oligomers, which results in the activation of downstream signaling cascades that determine cell behavior (2, 4, 5, 6). Dysregulation of RTK activity has been linked to many human diseases, including most cancers (2, 3, 7, 8). Thus, RTKs are promising drug targets, and a number of RTK inhibitors are already used in the clinic (9, 10, 11, 12).

The single transmembrane (TM) helix embedded in the plasma membrane connects the extracellular and intracellular portions of an RTK and may thus play a critical role in signal transduction across the plasma membrane. Work in the past two decades has demonstrated that contacts between TM helices can contribute to the overall stability of RTK dimers and oligomers (13, 14, 15, 16). Furthermore, RTK TM helices have been proposed to interact *via* two specific dimerization motifs characteristic of either inactive or active RTK states, suggesting a role of the TM helix in RTK activation (13, 17, 18, 19, 20, 21). The switch from one well-defined TM dimer structure to the other, occurring upon ligand binding, could be a mechanism enabling the transmission of information about the presence of a bound ligand to the kinase domain (22). However, this concept of a “TM dimer switch” has been mainly supported by experimental data obtained with isolated TM helices and by computational modeling (19, 20, 21, 23). On the other hand, the consequences of TM helix mutagenesis on the function of full-length RTKs have been difficult to interpret based simply on the TM dimer switch model (24, 25).

We seek to understand the role of the TM helix in the activation of EphA2, an RTK that plays important roles in cancer, inflammation, atherosclerosis, and infections (26, 27, 28). The architecture of EphA2 includes an extracellular region (with an N-terminal ligand-binding domain, a cysteine-rich region, and two fibronectin type III domains) (29, 30), a single TM helix, a flexible juxtamembrane segment of ∼50 amino acids, and a tyrosine kinase domain (29, 30). The EphA2 intracellular portion also includes a sterile alpha motif (SAM) domain and a short C-terminal tail. Interactions between EphA2 molecules in the plasma membrane are complex since EphA2 can form not only dimers but also higher order oligomers of different sizes (29, 30, 31). Oligomerized EphA2 molecules phosphorylate each other on tyrosines in the juxtamembrane segment, the kinase domain, and the SAM domain (32). Tyrosine phosphorylation promotes EphA2 kinase activity and downstream signaling that controls cell morphology, adhesion, migration, proliferation, and survival (33, 34, 35, 36, 37).

The structure of the dimeric EphA2 TM helix, embedded in lipid bicelles mimicking the plasma membrane, has been solved by NMR (19). These studies utilized a peptide that includes EphA2 residues 523 to 563, encompassing a short N-terminal hydrophilic segment (corresponding to the end of the second fibronectin type III domain and an extracellular seven amino acid linker), the hydrophobic membrane-embedded sequence of the TM helix, and the HRRRK stop-transfer sequence representing the positively charged N-terminal portion of the juxtamembrane segment. In the bicelles, the EphA2 TM helices interact *via* an extended “heptad repeat (HR)” motif that includes residues G539, A542, and G553 (19). Mutagenesis studies suggest that a shorter segment, comprising EphA2 residues 531 to 563 including the TM helix and the N-terminal portion of the juxtamembrane segment, dimerizes in cells *via* a different “glycine zipper (GZ)” interface involving residues G540 and G544 (23). These and other studies of the isolated EphA2 TM helix have been interpreted on the basis of the ligand-induced “TM dimer switch” model, which proposes a switch in the conformation of dimerized TM helices from a discrete inactive conformation involving the HR interface to a different active conformation involving the GZ interface (18, 20). However, the relevance of this model to the behavior of full-length EphA2 in the plasma membrane is unknown.

EphA2 can be differentially activated by multiple ligands. The ligand most widely used to activate EphA2 is the engineered dimeric ephrinA1-Fc (32, 38), a chimeric protein composed of ephrinA1 fused to an antibody Fc region. EphrinA1-Fc potently promotes EphA2 kinase-dependent signaling (32, 39). The endogenous form of ephrinA1 is anchored on the cell surface by a glycosylphosphatidylinositol linkage, but can also be released in a monomeric form (m-ephrinA1) that can also activate EphA2 (40, 41). Studies of these two ligands, and engineered peptide ligands, have revealed that EphA2 ligands do not always cause substantial EphA2 kinase activation (41, 42). For instance, the small monomeric peptide ligand YSA (YSAYPDSVPMMSGSGSK) activates EphA2 only very weakly (41, 42). Even in the absence of ligand, EphA2 can be phosphorylated in HEK293T cells (43). Further, there are differences in the sizes of the oligomers forming in response to the different ligands, as determined by Fluorescence Intensity Fluctuations (FIF) spectrometry (44). In the absence of a ligand and when the YSA peptide is bound, the most abundant EphA2 oligomer detected is a dimer, while binding of m-ephrinA1 or ephrinA1-Fc shifts the oligomer distribution to higher EphA2 oligomer sizes. However, recently published pulsed-interleaved excitation fluorescence cross-correlation spectroscopy (PIE-FCCS) experiments show that EphA2 can associate into oligomers even in the absence of ligand (45). Taken together, these published data suggest that EphA2 can exist in heterogeneous populations of dimers and oligomers, with the ligands inducing the fusion of EphA2 oligomers into larger ones. Given this complex behavior, it is not clear if and how “the TM switch model” applies to EphA2 activation in the plasma membrane.

Here we investigate how mutations in the HR and GZ interfaces of the EphA2 TM helix affect the dimerization/oligomerization of EphA2 molecules in cells in the absence of ligand and the presence of ephrinA1-Fc, m-ephrinA1, and the YSA peptide. Given the complex oligomerization behavior of EphA2, we used two fluorescent techniques (Förster Resonance Energy Transfer (FRET) and FIF spectrometry) to understand the effects of the mutations on different aspects of the oligomerization process.

## Results

We first characterized the homo-association of EphA2 wild-type, the G539I/A542I/G553I HR mutant, and the G540I/G544I GZ mutant in the plasma membrane of HEK293T cells using FRET. The mutations were introduced in full-length EphA2 labeled at the C-terminus with mTurquoise (mTurq, the donor) or enhanced yellow fluorescent protein (eYFP, the acceptor), attached *via* a (GGS)_5_ flexible linker. We have shown that these fluorescent proteins do not perturb EphA2 autophosphorylation (46).

We used a quantitative FRET technique termed Fully Quantified Spectral Imaging FRET, which involves the acquisition of complete FRET and acceptor spectra using a two-photon microscope (47). This technique employs an assumption-free, fully resolved system of equations to calculate (i) the donor concentrations, (ii) the acceptor concentrations, and (iii) the FRET efficiencies in hundreds of live cells (47). We used transient transfections to vary EphA2 expression over a broad range, from ∼100 to >6000 receptors per square micron, and we combined data from at least 100 cells to obtain FRET interaction curves.

**Figure 1:**
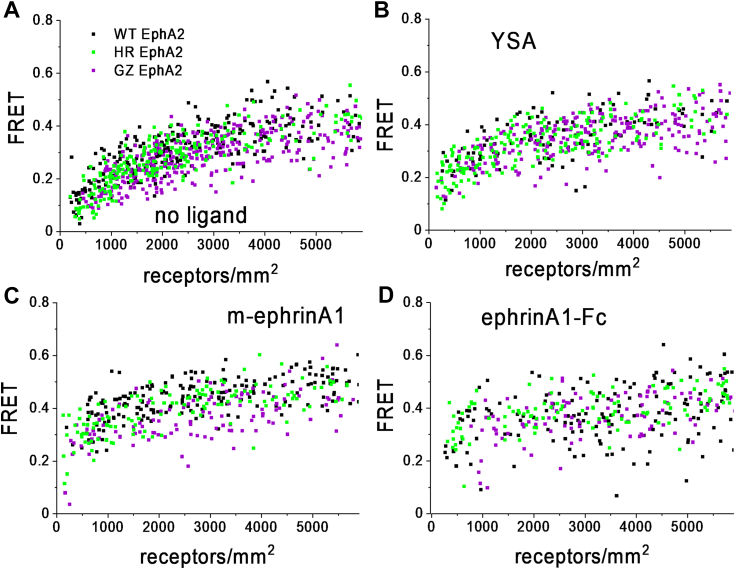
**FRET data for EphA2wild-type(*black*), the EphA2 HR mutant (*****green*****), and the EphA2 GZ mutant (*purple*).** FRET efficiency is shown as a function of EphA2 concentrations for either no added ligand (*A*), 50 μM YSA (*B*), 200 nM m-ephrinA1 (*C*), or 50 nM ephrinA1-Fc (*D*).

Experiments to compare EphA2 wild-type with the HR and GZ mutants were performed in the absence of ligand as well as in the presence of saturating/near saturating concentrations of ephrinA1-Fc (50 nM), m-ephrinA1 (200 nM) or YSA peptide (50 μM) (41) to ensure that most EphA2 receptors were ligand bound, and therefore that essentially only liganded dimers/oligomers contributed to the FRET signal. The measured FRET efficiencies were plotted as a function of the total EphA2 receptor (EphA2-mTurq + EphA2-eYFP) concentrations (Fig. 1), with each data point corresponding to one cell.

**Figure 2:**
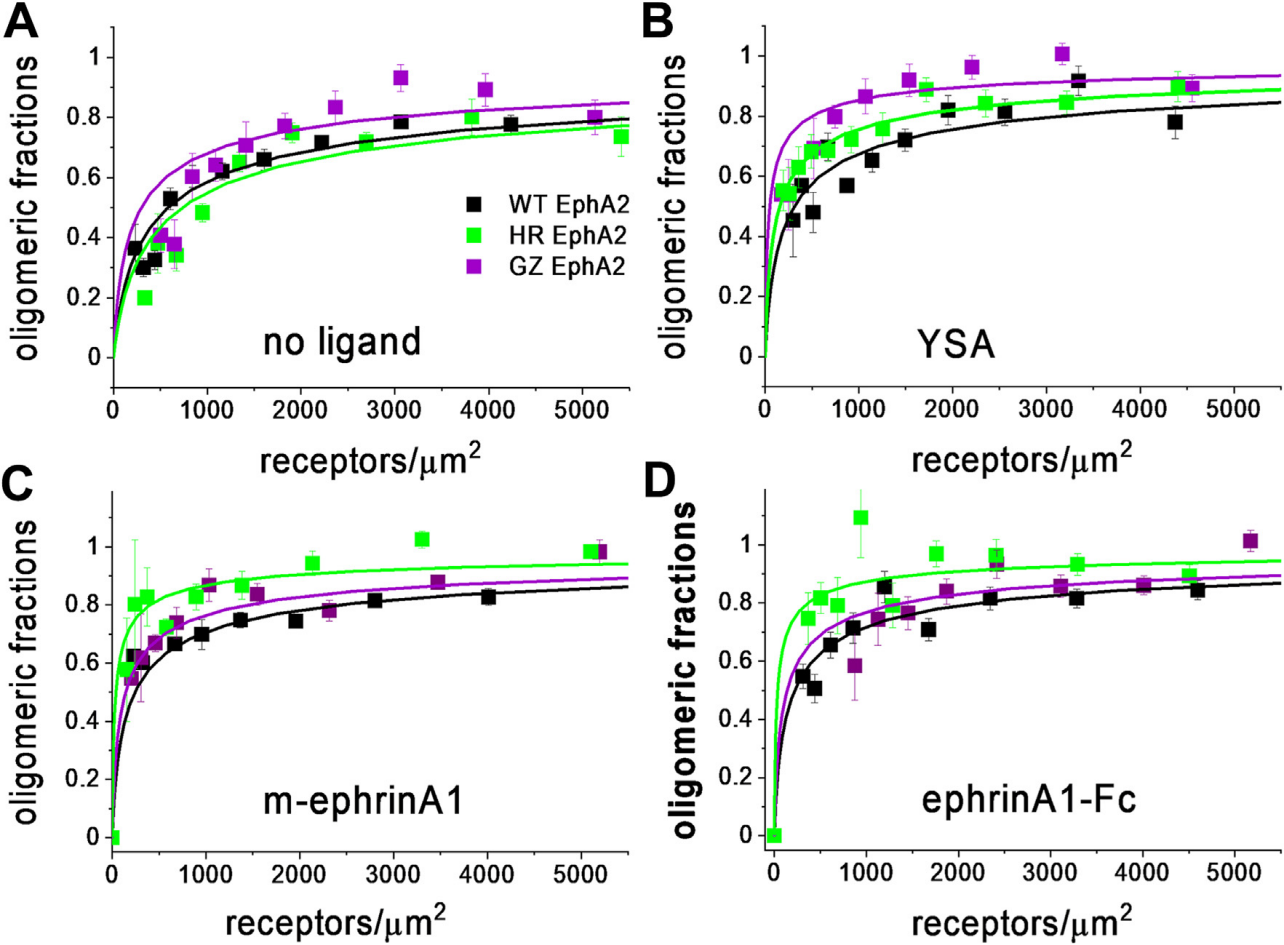
**Association curves from the FRET data, showing the fraction of oligomerized EphA2 as a function of EphA2 concentration.** Plots shown are for either no added ligand (*A*), 50 μM YSA (*B*), 200 nM m-ephrinA1 (*C*), or 50 nM ephrinA1-Fc (*D*). The association model (Equation 3) was fitted to all FRET data and the *solid lines* are constructed for the best-fit values of K_diss_ reported in Table 2. The symbols are averaged experimental data.

The single-cell FRET data were fitted using (Equation 3) and the effective equilibrium dissociation constant K_diss_ was calculated using (Equation 4). K_diss_ represents the receptor concentration for which half of the receptors are monomeric and half form dimers or higher order oligomers. This recently introduced thermodynamic constant reports on the stability of the oligomers, independently from oligomer sizes (48). The best-fit K_diss_ values for the wild-type and the two mutants (Table 1) were used to construct the oligomerization curves in Figure 2. The data analysis suggested that the HR mutant has a lower K_diss_ than EphA2 wild-type in the case of m-ephrinA1 and ephrinA1-Fc, but there are no statistically significant differences in the cases of no ligand and YSA (Tables 1 and 2). This indicates that the HR mutation stabilizes EphA2 oligomers bound to m-ephrin and ephrinA1-Fc. The GZ mutation has no statistically significant effect on EphA2 oligomer stability under any condition.

**Table 1:** Best-fit parameters for the FRET experiments

EphA2	K_diss_ (rec/μm^2^)	Ẽ	N
no ligand			
WT	581 ± 128	0.47 ± 0.02	255
HR	731 ± 293	0.40 ± 0.04	236
GZ	297 ± 170	0.33 ± 0.02	359
YSA			
WT	310 ± 140	0.60 ± 0.04	105
HR	155 ± 59	0.42 ± 0.02	193
GZ	49 ± 43	0.40 ± 0.02	257
m-ephrinA1			
WT	243 ± 71	0.66 ± 0.02	226
HR	40 ± 30	0.52 ± 0.02	187
GZ	144 ± 73	0.54 ± 0.03	152
ephrinA1-Fc			
WT	220 ± 84	0.59 ± 0.02	196
HR	36 ± 27	0.50 ± 0.02	144
GZ	137 ± 96	0.54 ± 0.03	156

K_diss_, dissociation constant; Ẽ, intrinsic FRET; N, number of individual cells analyzed in each experiment.

**Table 2:** Statistical analyses of the EphA2 FRET best-fit parameters

The *p*-values for K_diss_ were determined using two-sample t-tests with Welch’s correction for unequal SDs. The *p*-values for Ẽ were calculated with ANOVA in Prism followed by Tukey’s multiple comparison test. Comparisons with 0.001<*p*< 0.05 are colored light grey and comparisons with *p* ≤ 0.001 are colored dark grey.

In addition to K_diss_, the FRET experiments also yield a structural parameter called intrinsic FRET, Ẽ, which depends on the distance, dynamics, and orientation of the fluorescent proteins in the oligomers. A change in Ẽ due to a mutation indicates that the mutation induces a structural change in the EphA2 oligomers. Here we see that the HR mutation has a highly statistically significant effect on Ẽ, compared to WT, in the presence of YSA or m-ephrinA1 and also significantly affects Ẽ in the presence of the dimeric ligand ephrinA1-Fc. The GZ mutation has a highly statistically significant effect, compared to WT, in the absence of ligand and in the presence of YSA and m-ephrinA1 but not in the presence of ephrinA1-Fc. In all cases, Ẽ values are smaller for the EphA2 mutants than for EphA2 wild type, indicating a larger separation between the fluorophores in the mutant oligomers as compared to wild-type.

**Figure 3:**
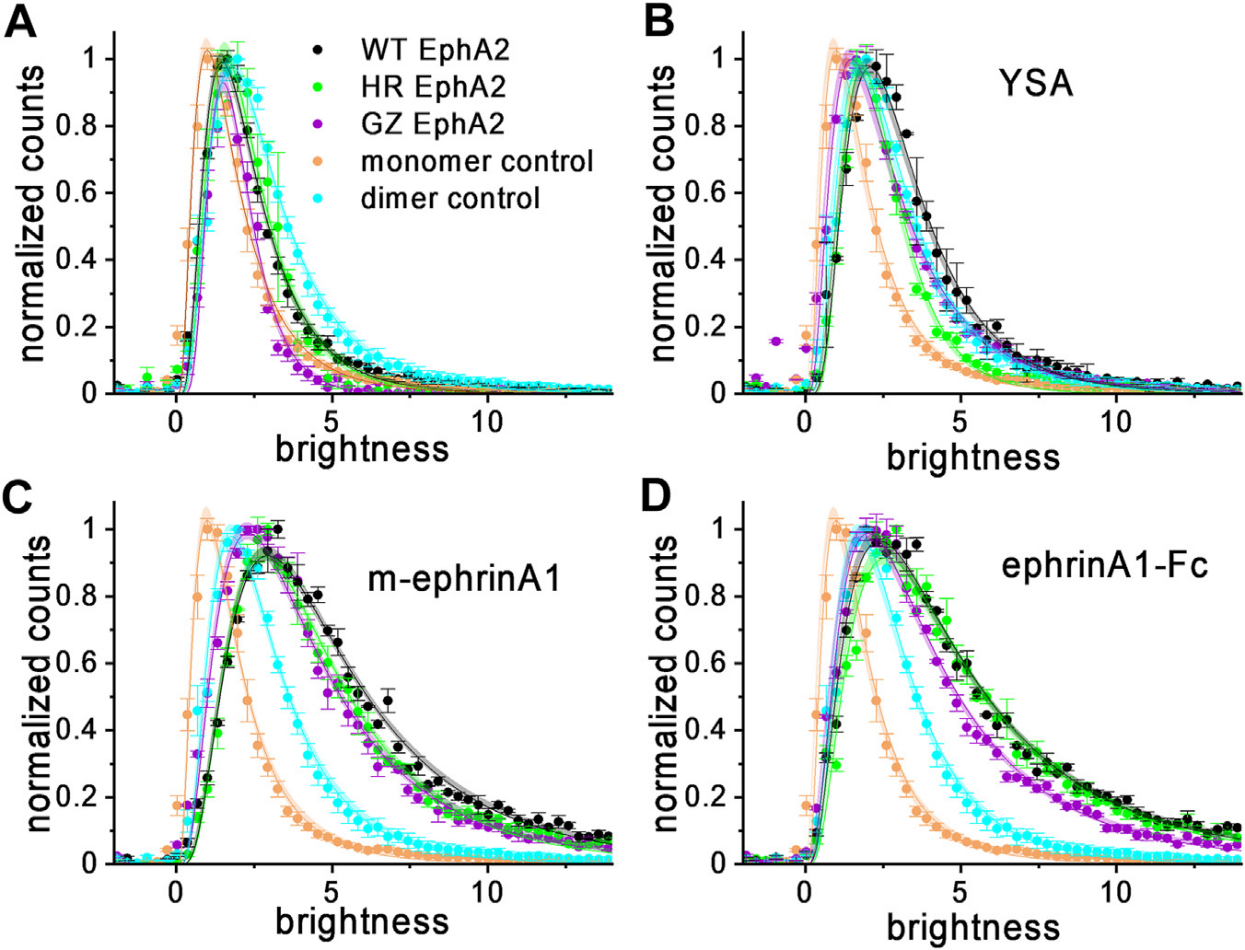
**FIF data for EphA2wild-type(*black*), the EphA2 HR mutant (*green*), and the EphA2 GZ mutant (*purple*).** Shown are molecular brightness distributions for EphA2 with no added ligand (*A*), 50 μM YSA (*B*), 200 nM m-ephrinA1 (*C*) and 50 nM ephrinA1-Fc (*D*) as averages and standard errors. The monomer control LAT (*orange*) and the dimer control E-cadherin (*turquoise*) are also shown in each panel.

To gain further insight into the effects of the HR and GZ mutations on EphA2 dimerization/oligomerization, we used FIF spectrometry. FIF measures molecular brightness, which is known to scale with oligomer size (49). For the FIF experiments, HEK293T cells were transiently transfected with plasmids encoding wild-type and mutant EphA2-eYFP as well as LAT-eYFP as a monomer control (50, 51) and E-cadherin-eYFP as a dimer control (52). Following the expression of the fluorescent proteins, the plasma membrane in contact with the substrate was imaged in a confocal microscope. Molecular brightness values were calculated in small regions of the plasma membrane (15 × 15 pixels) according to (Equation 5), as described (49), and values from thousands of such regions were used to construct a histogram (Fig. 3).

In the presence of ephrinA1-Fc and m-ephrinA1, EphA2 wild-type and mutant brightness distributions are shifted to higher brightness compared to the cases of no ligand and YSA peptide (Fig. 3), indicating the formation of higher order oligomers in accordance with published work (44). The effects of the mutations were assessed by fitting the different FIF distributions with a log-normal function, which is characterized by two best-fit parameters (μ and ω, see (Equation 6)). These two parameters are used to calculate the mean of the log-normal distributions (Table 3), enabling comparisons of the FIF data to determine if there are statistically significant differences in oligomer sizes (Table 4). Comparing the means of the FIF brightness distributions shows that the HR mutation significantly shifts the FIF distributions to lower brightness values compared to EphA2 wild-type in the presence of YSA and m-ephrinA1, indicating smaller oligomer sizes (Tables 3 and 4). On the other hand, the GZ mutant shows significantly smaller oligomer sizes than EphA2 wild-type in the absence of ligand and in the presence of all three ligands (Tables 3 and 4).

**Table 3:** Best-fit parameters for the FIF distributions

Protein	μ	ω	Mean
LAT	0.49 ± 0.02	0.71 ± 0.01	2.10 ± 0.04
E-cadherin	1.05 ± 0.02	0.70 ± 0.02	3.65 ± 0.08
no ligand - EphA2 WT	0.74 ± 0.01	0.60 ± 0.01	2.52 ± 0.03
no ligand - EphA2 GZ	0.61 ± 0.01	0.45 ± 0.01	2.04 ± 0.02
no ligand - EphA2 HR	0.76 ± 0.02	0.56 ± 0.01	2.51 ± 0.04
YSA - EphA2 WT	1.06 ± 0.01	0.58 ± 0.01	3.42 ± 0.04
YSA - EphA2 HR	0.84 ± 0.01	0.49 ± 0.01	2.62 ± 0.02
YSA - EphA2 GZ	0.90 ± 0.01	0.71 ± 0.01	3.16 ± 0.05
m-ephrinA1 - EphA2 WT	1.52 ± 0.01	0.67 ± 0.01	5.74 ± 0.07
m-ephrinA1 - EphA2 HR	1.42 ± 0.01	0.63 ± 0.01	5.04 ± 0.05
m-ephrinA1 - EphA2 GZ	1.34 ± 0.01	0.71 ± 0.01	4.93 ± 0.08
ephrinA1-Fc - EphA2 WT	1.48 ± 0.01	0.78 ± 0.01	5.97 ± 0.09
ephrinA1-Fc - EphA2 HR	1.50 ± 0.02	0.74 ± 0.01	5.86 ± 0.11
ephrinA1-Fc - EphA2 GZ	1.29 ± 0.01	0.77 ± 0.01	4.88 ± 0.07

μ and ω are the two best-fit parameters of the log-normal distribution (Equation 6).

They are used to calculate the mean according to (Equation 7).

**Table 4:** Statistical analysis of FIF means

Statistical analysis of FIF means was performed with ANOVA followed by Tukey’s multiple comparison test. *p* < 0.0001, dark grey, indicates very high statistical significance.

## Discussion

EphA2 has been long known to form oligomers in the plasma membrane, and not just classical dimers (30). The distribution of oligomer sizes is heterogeneous, especially for EphA2 bound to m-ephrinA1 and ephrinA1-Fc (44). In the absence of ligands and in the presence of YSA, the distribution is characterized by lower oligomer sizes, with dimers being prevalent (44). However, oligomers larger than dimers have been observed even in the absence of ligands (45). This heterogeneity in oligomer sizes makes comparisons of association behaviors of EphA2 variants challenging, as both stability and oligomer size may be affected by mutations. Recent methodological developments, however, enable the study of the effect of EphA2 TM helix mutations on EphA2 oligomerization in the plasma membrane. Here we used FRET to probe for changes in the EphA2 effective dissociation constant K_diss_ and in intrinsic FRET, a structure-sensitive parameter, due to TM helix mutations. We also used FIF to assess changes in the distribution of EphA2 oligomer sizes due to mutations. We mutated residues in the TM helix of EphA2 that have been previously reported to mediate contacts between the isolated TM helices in an NMR structure (19) as well as contacts identified in isolated TM helices in the plasma membrane of cells (18, 23). The interfaces we mutated are believed to be important in the context of the TM helix switch model for EphA2. Table 5 summarizes the effects of the mutations on the three parameters measured, K_diss_ (a measure of oligomer stability), Ẽ (a measure of oligomer structure), and brightness (a measure of oligomer size).

**Table 5:** Summary of results

“+” indicates statistical significance (0.001<*p* < 0.05, light grey) and “+++” indicates very high statistical significance (*p* ≤ 0.001, dark grey). “–“ indicates *p* >0.05.

According to the TM dimer switch model, we would expect effects due to only one of the mutated interfaces in the absence of ligand or the presence of YSA and effects due to only the other mutated interface in the cases of m-ephrinA1 and ephrinA1-Fc. This expectation is based on published functional characterization of EphA2, which has demonstrated that m-ephrinA1 and ephrinA1-Fc activate EphA2 very efficiently, while the unliganded and YSA-bound EphA2 receptor is inefficiently auto-phosphorylated, and is incapable of strong activation of downstream signaling (41). Our results are not consistent with this expectation (Table 5), indicating that the TM dimer switch model does not apply to EphA2. The only case in which a mutation does not change any of the three EphA2 oligomerization parameters is the HR mutation in the absence of a ligand. This suggests that the HR interface is not involved in the stabilization of EphA2 unliganded dimers/oligomers, pointing to the significance of the GZ interface in the stabilization of EphA2 unliganded dimers/oligomers. However, both the HR and GZ interfaces play a role in the stabilization of ligand-bound EphA2 oligomers.

Functional studies of mutations engineered to destabilize either the HR or the GZ interface in full-length EphA2 also did not support the ligand-induced TM dimer switch model (18). Although mutations in the HR and GZ motifs increase and decrease EphA2 tyrosine phosphorylation, respectively, as compared to the wild-type receptor, the effects of the mutations were the same in the presence and absence of ligand (18). Other recent findings further raise the possibility that the specific arrangement of the TM helices in EphA2 dimers and oligomers may not be critical for receptor activation. For instance, the deletion of most of the juxtamembrane segment (residues 565–606) severely compromises the ability of EphA2 molecules to cross-phosphorylate on the remaining tyrosine residues (including Y772 in the activation loop of the kinase domain and Y930 in the SAM domain) (41). This suggests that the flexible juxtamembrane sequence, connecting the TM helix with the kinase domain, allows differential positioning of EphA2 molecules for cross-phosphorylation on different tyrosine residues. The flexibility of this segment argues against tight structural coupling between the TM helix and the kinase domain and thus against a major impact of TM dimeric arrangements on EphA2 tyrosine phosphorylation and activation.

It is possible that the TM dimer switch model does not apply to EphA2 because EphA2 forms oligomers, and not just dimers. Thus, the TM domains may interact as oligomers such as trimers or consecutively engage different interfaces. Notably, functions other than control of EphA2 activation have been proposed for the EphA2 TM helix, such as the regulation of EphA2 localization at epithelial cell-cell junctions (53).

An alternative way to interpret our results is to hypothesize that the TM helices in EphA2 dimers and oligomers can interact promiscuously and transiently, *via* different non-unique interfaces. The existence of an ensemble of TM helix dimer interfaces utilized within an RTK dimer could provide an explanation of our results since mutagenesis could switch the relative preference to interfaces that are more stable. The existence of an ensemble of TM helix interfaces populated within an RTK dimer or oligomer could be one of the reasons explaining why the RTK TM helices have remained unresolved in cryo-EM structures. It can be speculated that this plasticity in TM helix contacts may augment the overall conformational space that is accessible to the EphA2 intracellular region. This could facilitate the formation of diverse intracellular arrangements of EphA2 dimers and oligomers to ensure their ability to cross-phosphorylate and/or phosphorylate substrate proteins. Thus, the TM helices may be critical for the ability of EphA2 to trigger diverse downstream signaling cascades, but not in the simple manner predicted by the TM dimer switch model.

While our study shows that EphA2 association is not consistent with the TM dimer switch model, it also reveals that mutagenesis of the TM helices affects EphA2 stability, structure, and oligomer size, as summarized in Table 5. Thus, the interactions between the helices contribute to the self-association of EphA2 in the plasma membrane, consistent with the published functional studies that also revealed an effect (18). The exact mechanism appears more complex and perhaps more subtle than proposed by the TM dimer switch model, and is yet to be uncovered.

## Experimental procedures

### Plasmid constructs

The EphA2 plasmid in the pcDNA3.1(+) vector encodes for human EphA2 tagged at the C-terminus with a fluorescent protein (either eYFP or mTurquoise) *via* a 15 amino acid GGS_5_ linker (43). The glycine zipper (GZ, G540I, G544I) and heptad repeat (HR, G539I, A542I, G553I) variants were cloned using the QuikChange II Site-Directed Mutagenesis Kit according to the manufacturer’s instructions (Agilent Technologies, #200523). All plasmids were sequenced to confirm the correct sequences (Genewiz).

### Cell culture and transfection

HEK293T cells were purchased from the American Type Culture Collection. They were tested for *mycoplasma* and were free of contamination. The cells were cultured in Dulbecco’s modified eagle medium (Gibco, #31600034) supplemented with 10% fetal bovine serum (HyClone, #SH30070.03), 20 mM D-Glucose and 18 mM sodium bicarbonate at 37 °C in a 5% CO_2_ environment.

24 h prior to transfection, cells were seeded in 35 mm glass coverslip, collagen-coated Petri dishes (MatTek, P35GCOL-1.5–14-C) at a density of 2.5∗10^5^ cells per dish to reach ∼70% confluency at the day of the experiment. For transfection, Lipofectamine 3000 (Invitrogen, #L3000008) was used according to the manufacturer’s protocol. Single transfections were performed using 1 to 3 ug plasmid DNA. Co-transfections were performed with 1 to 4 ug total plasmid DNA in a 1:3 donor:acceptor ratio. 12 h after transfection the cells were rinsed twice with phenol-red free, serum-free starvation media and then serum starved for at least 12 h. For experiments with added ligand, the starvation medium was supplemented with 0.1% BSA to coat the wall of the dishes.

### Two photon microscopy

Before imaging, HEK293T cells were subjected to reversible osmotic stress by replacing the serum-free medium with a 37 °C, 1:9 serum-free medium:diH_2_O, 25 mM HEPES solution. In cells. the plasma membrane is normally highly ruffled and its topology in microscope images is virtually unknown (47). The reversible osmotic stress eliminates these wrinkles and allows to convert effective 3D protein concentrations into 2D receptor concentrations (47). In experiments with ligands, the swelling media was supplemented with 200 nM monomeric EprhinA1 (Novoprotein, #CA70), 50 nM dimeric EphrinA1-Fc (R&D Systems, #602-A1-200), or 50 μM of the engineered peptide ligand YSA (YSAYPDSVPMMSGSGSK). The cells were allowed to equilibrate for 10 min at room temperature. Fully Quantified Spectral Imaging FRET, a quantitative fluorescent microscopy imaging and analysis technique, was used to measure donor (EphA2-mTurquoise) concentrations, acceptor (EphA2-eYFP) concentrations, and FRET efficiencies in individual cells (47). Images of cells (100 to 350 cells per condition, see Table 2) were acquired using a two-photon microscope equipped with the OptiMiS spectral imaging system (Aurora Spectral Technologies). Two scans were performed for every cell– a FRET scan (λ_1_=840 nm) in which the donor (mTurquoise) is primarily excited and an acceptor scan (λ_2_=960 nm) in which the acceptor (eYFP) is primarily excited. The output of each scan is composed of 300 × 440 pixels, where every pixel contains a full fluorescence spectrum in the range of 420 to 620 nm. Images of cells were acquired for up to 2 h. Micron-sized regions of the plasma membrane that are not in contact with adjacent cells were analyzed to obtain the donor concentration, the acceptor concentration, and the FRET efficiencies as described (Equations (1), (2), (3) in ref (48)).

We calculate the effective dissociation constant by fitting a monomer-oligomer model to the FRET data, given by the following reaction scheme:

(1) n[m]⇌[oligomer]

Here *n* is the best estimate of the average oligomer order. The dissociation constant for this reaction is defined as:

(2) $$K_{D}^{oligomer}=\frac{{\left[m\right]}^{n}}{\left[oligomer\right]}$$

Details about the calculation of $K_{D}^{oligomer}$ are given in published work (48). Briefly, we fit the FRET data to the following equation

(3) $$FRET=\frac{\left[T\right]-\left[m\right]\left(\left[T\right],K_{D}^{oligomer},n\right)}{\left[T\right]x_{D}}\;\sum_{k=1}^{n-1}\frac{k\left(n\;-\;k\right)\tilde{E}}{1\;+\;\left(n\;-\;k-1\right)\tilde{E}}\left(\begin{array}{c} n\\ k \end{array}\right)x_{D}^{k}x_{A}^{n-k}$$

Here $x_{D}\;\&\;x_{A}$ are the fraction of donors and acceptors, and $\tilde{E}$ is the so-called “intrinsic FRET” which depends on the distance between the fluorophores in the oligomer. [T] is the total receptor concentration (sum of donor- and acceptor-labeled EphA2). The parameters $x_{A}$, $x_{D}$, [T], and FRET efficiency (after correction for proximity (54, 55)) are measured in the experiment for each cell. $K_{D}^{oligomer}$ and $\tilde{E}$ are the unknowns, and their best-fit values are determined in the fit.

We then use this dissociation constant, which has units of (receptors/μm^2^)^*n-1*^, to calculate an effective dissociation constant with units of EphA2 concentration in the membrane according to.

(4) $$K_{D_{eff}}^{oligomer}=\left[T^{*}\right]=2{\left(\frac{K_{D}^{oligomer}}{n}\right)}^{\frac{1}{n-1}}$$

As shown previously, $K_{D_{eff}}^{oligomer}$ does not depend on the value of *n* chosen for the fit (48). Further, $K_{D_{eff}}^{oligomer}$ has a very well defined physical meaning as it is the concentration for which 50% of the receptors are associated into oligomers and thus active and 50% are monomeric and thus inactive.

### Fluorescence Intensity Fluctuations spectroscopy and analysis

FIF experiments were performed with a TCS SP8 confocal microscope (Leica Biosystems, Wetzlar, Germany) equipped with a HyD hybrid detector. Images (1024 × 1024, 12 bit) of plasma membranes of cells expressing EphA2-eYFP were acquired in photon counting mode with a scanning speed of 20 Hz and a 488 nm diode laser excitation. The emission spectra of eYFP were collected from 520 to 580 nm.

eYFP was excited using a 488 nm diode laser at 0.1% to avoid photobleaching, at a scanning speed of 20Hz. Cells were subjected to osmotic stress with a hypoosmotic media of 75% water. This swelling minimizes the effect of ruffles, folds, invaginations, or other irregularities in the plasma membrane, while also preventing endocytosis of the receptor.

A total of ∼100 to 150 cells were imaged and analyzed. A large region in the plasma membrane was selected for each cell and was then divided into segments of 15 × 15 (225 pixels^2^) as described (49), yielding a total of ∼10,000 segments per ligand. Histograms of pixel intensities were constructed. Errors were calculated using a bootstrapping methods, where cells were grouped into three random pools. The histograms were averaged and the standard error was calculated.

The histograms were fitted with a Gaussian function, yielding two parameters: <*I*_*segment*_>, the center of the Gaussian, and *σ*_*segment*_, the width of the Gaussian for each segment.

The molecular brightness of each segment *ε*_*segment*_ was calculated as:

(5) $$\varepsilon_{segment}=\frac{\sigma_{segment}^{2}}{\left\langle I_{segment}\right\rangle }\;-\;1$$

The brightness values from thousands of segments were binned and used to generate a histogram.

These brightness distributions were fitted using Origin Lab with a log-normal function given by:

(6) $$y=\frac{A}{\omega x\sqrt{2\pi }}\;\exp\left(-\;\frac{{\left(\ln\left(x\right)-\mu \right)}^{2}}{2\omega^{2}}\right)$$

Here μ is the mean of the respective ln(x) Gaussian distribution and ω is the width of the distribution. These two parameters were used to calculate the mean of the log-normal distribution according to:

(7) $$mean=\exp\left(\mu +\left(\frac{\omega^{2}}{2}\right)\right)$$

### Statistical analysis

The means of the brightness distributions (from FIF experiments) and the $\tilde{\;E}$ values (from FRET experiments) were compared using ANOVA in Prism. We compared K_diss_ for wild-type EphA2 and the two mutants using two-sample t-tests. Because F-tests for unequal variances showed highly significant differences between SDs in this case, we used Welch’s corrected t-tests for unequal variances.

## Data availability

All data are included in the article.

## Conflict of interest

The authors declare that they have no known competing financial interests or personal relationships that could have appeared to influence the work reported in this paper.

## References

[1] Blume-Jensen P., Hunter T. (2001). Oncogenic kinase signalling. Nature.

[2] Lemmon M.A., Schlessinger J. (2010). Cell signaling by receptor tyrosine kinases. Cell.

[3] Schlessinger J. (2014). Receptor tyrosine kinases: legacy of the first two decades. Cold Spring Harb. Perspect. Biol..

[4] Arteaga C.L., Engelman J.A. (2014). ERBB receptors: from oncogene discovery to basic science to mechanism-based cancer therapeutics. Cancer Cell.

[5] Belov A.A., Mohammadi M. (2013). Molecular mechanisms of fibroblast growth factor signaling in physiology and pathology. Cold Spring Harb. Perspect. Biol..

[6] Wagner M.J., Stacey M.M., Liu B.A., Pawson T. (2013). Molecular mechanisms of SH2- and PTB-domain-containing proteins in receptor tyrosine kinase signaling. Cold Spring Harb. Perspect. Biol..

[7] Neben C.L., Lo M., Jura N., Klein O.D. (2019). Feedback regulation of RTK signaling in development. Dev. Biol..

[8] Kumar R., George B., Campbell M.R., Verma N., Paul A.M., Melo-Alvim C. (2020). HER family in cancer progression: from discovery to 2020 and beyond. Adv. Cancer Res..

[9] Attwood M.M., Fabbro D., Sokolov A.V., Knapp S., Schioth H.B. (2021). Trends in kinase drug discovery: targets, indications and inhibitor design. Nat. Rev. Drug Discov..

[10] Tebbutt N., Pedersen M.W., Johns T.G. (2013). Targeting the ERBB family in cancer: couples therapy. Nat. Rev. Cancer.

[11] Yamaoka T., Kusumoto S., Ando K., Ohba M., Ohmori T. (2018). Receptor tyrosine kinase-targeted cancer therapy. Int. J. Mol. Sci..

[12] Ferguson F.M., Gray N.S. (2018). Kinase inhibitors: the road ahead. Nat. Rev. Drug Discov..

[13] Li E., Hristova K. (2010). Receptor tyrosine kinase transmembrane domains: function, dimer structure and dimerization energetics. Cell Adh. Migr..

[14] Del Piccolo N., Placone J., Hristova K. (2015). Effect of Thanatophoric Dysplasia type I mutations on FGFR3 dimerization. Biophys. J..

[15] Sarabipour S., Hristova K. (2016). Mechanism of FGF receptor dimerization and activation. Nat. Commun..

[16] Sarabipour S., Hristova K. (2016). Effect of the achondroplasia mutation on FGFR3 dimerization and FGFR3 structural response to fgf1 and fgf2: a quantitative FRET study in osmotically derived plasma membrane vesicles. Biochim. Biophys. Acta.

[17] Li E., Hristova K. (2006). Role of receptor tyrosine kinase transmembrane domains in cell signaling and human pathologies. Biochemistry.

[18] Sharonov G.V., Bocharov E.V., Kolosov P.M., Astapova M.V., Arseniev A.S., Feofanov A.V. (2014). Point mutations in dimerization motifs of the transmembrane domain stabilize active or inactive state of the EphA2 receptor tyrosine kinase. J. Biol. Chem..

[19] Bocharov E.V., Mayzel M.L., Volynsky P.E., Mineev K.S., Tkach E.N., Ermolyuk Y.S. (2010). Left-handed dimer of EphA2 transmembrane domain: helix packing diversity among receptor tyrosine kinases. Biophys. J..

[20] Stefanski K.M., Russell C.M., Westerfield J.M., Lamichhane R., Barrera F.N. (2021). PIP2 promotes conformation-specific dimerization of the EphA2 membrane region. J. Biol. Chem..

[21] Fleishman S.J., Schlessinger J., Ben-Tal N. (2002). A putative molecular-activation switch in the transmembrane domain of erbB2. Proc. Natl. Acad. Sci. U. S. A..

[22] Wilson K.J., Gilmore J.L., Foley J., Lemmon M.A., Riese D.J. (2009). Functional selectivity of EGF family peptide growth factors: implications for cancer. Pharmacol. Ther..

[23] Westerfield J.M., Sahoo A.R., Alves D.S., Grau B., Cameron A., Maxwell M. (2021). Conformational clamping by a membrane ligand activates the EphA2 receptor. J. Mol. Biol..

[24] Bocharov E.V., Mineev K.S., Pavlov K.V., Akimov S.A., Kuznetsov A.S., Efremov R.G. (2017). Helix-helix interactions in membrane domains of bitopic proteins: specificity and role of lipid environment. Biochim. Biophys. Acta.

[25] Bartzoka F., Gonzalez-Magaldi M., Byrne P.O., Callery N.I., Hristova K., Leahy D.J. (2022). Activity of EGFR transmembrane region variants indicates specific transmembrane dimers are not required for EGFR activity. Biochem. J..

[26] Xiao T., Xiao Y., Wang W., Tang Y.Y., Xiao Z., Su M. (2020). Targeting EphA2 in cancer. J. Hematol. Oncol..

[27] Wilson K., Shiuan E., Brantley-Sieders D.M. (2021). Oncogenic functions and therapeutic targeting of EphA2 in cancer. Oncogene.

[28] Riedl S.J., Pasquale E.B. (2015). Targeting the Eph system with peptides and peptide conjugates. Curr. Drug Targets.

[29] Seiradake E., Harlos K., Sutton G., Aricescu A.R., Jones E.Y. (2010). An extracellular steric seeding mechanism for Eph-ephrin signaling platform assembly. Nat. Struct. Mol. Biol..

[30] Himanen J.P., Yermekbayeva L., Janes P.W., Walker J.R., Xu K., Atapattu L. (2010). Architecture of Eph receptor clusters. Proc. Natl. Acad. Sci. U. S. A..

[31] Lechtenberg B.C., Gehring M.P., Light T.P., Horne C.R., Matsumoto M.W., Hristova K. (2021). Regulation of the EphA2 receptor intracellular region by phosphomimetic negative charges in the kinase-SAM linker. Nat. Commun..

[32] Barquilla A., Pasquale E.B. (2015). Eph receptors and ephrins: therapeutic opportunities. Annu. Rev. Pharmacol. Toxicol..

[33] Pasquale E.B. (2005). Eph receptor signalling casts a wide net on cell behaviour. Nat. Rev. Mol. Cell Biol..

[34] Pasquale E.B. (2008). Eph-ephrin bidirectional signaling in physiology and disease. Cell.

[35] Pasquale E.B. (2010). Eph receptors and ephrins in cancer: bidirectional signalling and beyond. Nat. Rev. Cancer.

[36] Lisabeth E.M., Falivelli G., Pasquale E.B. (2013). Eph receptor signaling and ephrins. Cold Spring Harb. Perspect. Biol..

[37] Boyd A.W., Bartlett P.F., Lackmann M. (2014). Therapeutic targeting of EPH receptors and their ligands. Nat. Rev. Drug Discov..

[38] Miao H., Burnett E., Kinch M., Simon E., Wang B. (2000). Activation of EphA2 kinase suppresses integrin function and causes focal-adhesion-kinase dephosphorylation. Nat. Cell Biol..

[39] Noberini R., de la Torre E.R., Pasquale E.B. (2012). Profiling Eph receptor expression in cells and tissues A targeted mass spectrometry approach. Cell Adh. Migr..

[40] Beauchamp A., Lively M.O., Mintz A., Gibo D., Wykosky J., Debinski W. (2012). EphrinA1 is released in three forms from cancer cells by matrix metalloproteases. Mol. Cell Biol..

[41] Gomez-Soler M., Gehring M.P., Lechtenberg B.C., Zapata-Mercado E., Ruelos A., Matsumoto M.W. (2022). Ligands with different dimeric configurations potently activate the EphA2 receptor and reveal its potential for biased signaling. iScience.

[42] Koolpe M., Dail M., Pasquale E.B. (2002). An ephrin mimetic peptide that selectively targets the EphA2 receptor. J. Biol. Chem..

[43] Singh D.R., Ahmed F., King C., Gupta N., Salotto M., Pasquale E.B. (2015). EphA2 receptor unliganded dimers suppress EphA2 pro-tumorigenic signaling. J. Biol. Chem..

[44] Zapata-Mercado E., Biener G., McKenzie D.M., Wimley W.C., Pasquale E.B., Raicu V. (2022). The efficacy of receptor tyrosine kinase EphA2 autophosphorylation increases with EphA2 oligomer size. J. Biol. Chem..

[45] Shi X., Lingerak R., Herting C.J., Ge Y., Kim S., Toth P. (2023). Time-resolved live-cell spectroscopy reveals EphA2 multimeric assembly. Science.

[46] Singh D.R., Ahmed F., Paul M.D., Gedam M., Pasquale E.B., Hristova K. (2017). The SAM domain inhibits EphA2 interactions in the plasma membrane. Biochim. Biophys. Acta Mol. Cell Res..

[47] King C., Stoneman M., Raicu V., Hristova K. (2016). Fully quantified spectral imaging reveals *in vivo* membrane protein interactions. Integr. Biol. (Camb).

[48] McKenzie D.M., Wirth D., Pogorelov T.V., Hristova K. (2023). Utility of FRET in studies of membrane protein oligomerization: the concept of the effective dissociation constant. Biophys. J..

[49] Stoneman M.R., Biener G., Ward R.J., Pediani J.D., Badu D., Eis A. (2019). A general method to quantify ligand-driven oligomerization from fluorescence-based images. Nat. Methods.

[50] Adámková L., Kvíčalová Z., Rozbeský D., Kukačka Z., Adámek D., Cebecauer M. (2019). Oligomeric architecture of mouse activating Nkrp1 receptors on living cells. Int. J. Mol. Sci..

[51] Paul M.D., Grubb H.N., Hristova K. (2020). Quantifying the strength of heterointeractions among receptor tyrosine kinases from different subfamilies: implications for cell signaling. J. Biol. Chem..

[52] Singh D.R., Ahmed F., Sarabipour S., Hristova K. (2017). Intracellular domain contacts contribute to ecadherin constitutive dimerization in the plasma membrane. J. Mol. Biol..

[53] Ventrella R., Kaplan N., Hoover P., Perez White B.E., Lavker R.M., Getsios S. (2018). EphA2 transmembrane domain is uniquely required for keratinocyte migration by regulating ephrin-A1 levels. J. Invest. Dermatol..

[54] King C., Raicu V., Hristova K. (2017). Understanding the FRET signatures of interacting membrane proteins. J. Biol. Chem..

[55] King C., Sarabipour S., Byrne P., Leahy D.J., Hristova K. (2014). The FRET signatures of non-interacting proteins in membranes: simulations and experiments. Biophys. J..

